# Pathogenic Roles of CXCL10 in Experimental Autoimmune Prostatitis by Modulating Macrophage Chemotaxis and Cytokine Secretion

**DOI:** 10.3389/fimmu.2021.706027

**Published:** 2021-09-29

**Authors:** Xiaoliang Hua, Shengdong Ge, Meng Zhang, Fan Mo, Ligang Zhang, Jiong Zhang, Cheng Yang, Sheng Tai, Xianguo Chen, Li Zhang, Chaozhao Liang

**Affiliations:** ^1^ Department of Urology, The First Affiliated Hospital of Anhui Medical University, Hefei, China; ^2^ Anhui Province Key Laboratory of Genitourinary Diseases, Anhui Medical University, Hefei, China; ^3^ The Institute of Urology, Anhui Medical University, Hefei, China; ^4^ Anhui Institute of Translational Medicine, Hefei, China

**Keywords:** chronic prostatitis, CXCL10, CXCR3, macrophage, inflammation

## Abstract

Chronic prostatitis and chronic pelvic pain syndrome (CP/CPPS) is an inflammatory immune disease characterized by intraprostatic leukocyte infiltration and pelvic or perineal pain. Macrophages play vital roles in the pathogenesis of CP/CPPS. However, the mechanisms controlling the activation and chemotaxis of macrophages in CP/CPPS remain unclear. This study aimed to investigate the roles of the CXCL10/CXCR3 pathway in the activation and chemotaxis of macrophages in CP/CPPS patients. The serums of CP/CPPS patients and healthy volunteers were collected and measured. Results showed that CXCL10 expression was significantly elevated and correlated with the severity of CP/CPPS patients. The experimental autoimmune prostatitis (EAP) model was generated, and adeno-associated virus and CXCR3 inhibitors were used to treat EAP mice. Immunofluorescence, flow cytometry, and Western blotting were used to analyze the functional phenotype and regulation mechanism of macrophages. Results showed that CXCL10 deficiency ameliorates EAP severity by inhibiting infiltration of macrophages to prostate. Moreover, CXCL10 could induce macrophage migrations and secretions of proinflammatory mediators *via* CXCR3, which consequently activated the downstream Erk1/2 and p38 MAPK signaling pathways. We also showed that prostatic stromal cell is a potential source of CXCL10. Our results indicated CXCL10 as an important mediator involved in inflammatory infiltration and pain symptoms of prostatitis by promoting the migration of macrophages and secretion of inflammatory mediators *via* CXCR3-mediated ERK and p38 MAPK activation.

## Introduction

Prostatitis is the most common urologic disease in men under 50 years old, accounting for 8% of urologist visits (1–3). Chronic prostatitis and chronic pelvic pain syndrome (CP/CPPS), classified as NIH category III prostatitis, is the most common type of prostatitis and represents more than 90% of all cases (2, 3). CP/CPPS is a major unmet medical problem, characterized by pelvic or perineal pain, irritating voiding symptoms, and sexual dysfunction, which showed a detrimental effect on sperm and had detrimental effects on the quality of life (4, 5). The etiology of CP/CPPS is unknown and the possible mechanisms include urine reflux-inducing trauma, dietary factors, pelvic floor dysfunction, hormone imbalance, and autoimmune imbalance (6, 7), while accumulating lines of evidence suggest an immune origin for CP/CPPS in patients and animal models (7–9).

Prostate histopathology for CP/CPPS patients showed diffuse distributions of leukocytes, predominantly dominated by T-lymphocytic and mononuclear cells (10, 11). Inflammation of the male genital tract for CP/CPPS patients was revealed by increased counts of leukocytes, mainly CD4+ T lymphocytes and macrophages in semen (5). The EAP model, a valid model for CP/CPPS, shares some histologic features with CP/CPPS in humans, which is defined by a florid intraprostatic leukocyte infiltrate, including CD4+ and CD8+ T cells, B cells, dendritic cell, and macrophages (12). Moreover, the intraprostatic inflammatory infiltrate in the EAP model is characterized by CD4+ T cells and macrophages (13, 14), suggesting its vital roles in the development of EAP (15). These cells could interact with other immune and resident cells, including epithelial and stromal cells by secreting cytokines and chemokines, leading to a boost in the production of proinflammatory cytokines and chemokines. The increased cytokines and chemokines in turn alter the activation or differentiation state of one or both of the cell types (16). However, the mechanisms controlling the activation and chemotaxis of CD4+ T cells and macrophages in CP/CPPS remain unclear. Previous studies support a crucial role of interferon-gamma (IFN-γ) in the pathogenesis of the disease in the EAP model (17, 18). IFN-γ-deficient mice showed a significantly decreased number of leukocyte infiltrates in the prostate in the EAP model, owing to impairing the abilities of T cells for homing to the prostate gland (18). Deficiency in IRF-1 or STAT-1 transcription factors involved in the IFN-γ signaling cascade made mice resistant to EAP, suggesting essential roles of IFN-γ signaling in disease induction (17). However, the molecules and specific mechanisms regulated by IFN-γ signaling remain to be explored.

In inflammatory conditions, chemokines including CXCL9, CXCL10, and CXCL11 could be secreted by a variety of cells in response to IFN-γ. These chemokines can interact with its common receptor CXCR3 to play a vital role in T-cell recruitment and immune response in a number of inflammatory and autoimmune diseases (19, 20). Although the role of CXCL10 in inflammation is appreciated, the expression of CXCL10 and its involvement in the pathogenesis of CP/CPPS have not been well elucidated. In the EAP model, studies showed that the expression of CXCR3 on specific T cells is essential for homing to the prostate gland (18). The roles of other immune cells expressing CXCR3 receptor in the pathogenesis of EAP, the ligands that are mainly involved, and the potential mechanisms remain unclear.

In the present study, we investigated the roles and specific mechanisms of CXCL10 in CP/CPPS patients. Our study showed high expressions of CXCL10 in CP/CPPS patients, and the expression of CXCL10 was associated with the severity of the pain and symptoms of the patient. CXCL10 could induce the secretion of important proinflammatory factors and chemotaxis of macrophages. In CXCL10-deficient mice, disease severity and inflammatory infiltration attenuated. We also showed that CXCL10 could activate extracellular signal-regulated kinase (Erk) and p38-mitogen-activated protein kinase (MAPK) signaling pathways by binding to CXCR3 to enhance the accumulation of inflammatory cells and secretion of inflammatory mediators. In addition, we also identified the origins of CXCL10, potentiating future targeted therapy.

## Materials and Methods

### Patient Recruitment

The present study was performed in accordance with the Declaration of Helsinki principles and was approved by the ethical committee of the First Affiliated Hospital of Anhui Medical University (P2021-03-13). Twenty-four patients with CP/CPPS were identified at the outpatient department of Dr. Liang at Anhui Medical University, China. All patients completed the National Institutes of Health Chronic Prostatitis Symptom Index (NIH-CPSI), a validated questionnaire designed to measure symptom severity (21, 22). Controls were healthy donors from a physical examination center of the First Affiliated Hospital of Anhui Medical University. Written informed consent was obtained from each participant. The inclusion criteria included first outpatient visit, no other complications, and no history of antibiotic therapy in the past 3 months. The exclusion criteria included age higher than 50 years.

### Mice and EAP Induction

Six- to 8-week-old male non-obese diabetic (NOD) mice were purchased from the Nanjing Biomedical Research Institute of Nanjing University (Nanjing, China). All the animals were housed in specific pathogen-free research animal facility of Anhui Medical University. All animal protocols were approved by the Committee for Animal Care and Use of the Animal Center of Anhui Medical University (LLSC20211058). The EAP mouse model was successfully induced as previously described (18, 23). Briefly, the prostate homogenate was obtained from the prostate of a Sprague–Dawley rat, and the supernatants were collected as prostate antigens (PAgs). Equal volumes of PAgs or saline solution were emulsified thoroughly in complete Freund’s adjuvant (CFA; Sigma‐Aldrich, St. Louis, MO). Mice were s.c. immunized in different locations, namely, bilateral hint footpads, the lower back, and the base of the tail, with PAgs (300 μg/mouse, EAP group) or phosphate buffer solution (control group) emulsified in CFA in a total volume of 150 μl/mouse. According to the experimental schedule (24), mice were immunized at days 0 and 28, and then were sacrificed at day 42. A small molecular weight antagonist of CXCR3, AMG487 (MedChem Express), was used to i.p. treat mice daily at 5 mg/kg (25). A 20% sulfobutylether-β-cyclodextrin (SBE-β-CD, HY-17031, MedChem Express) solution served as the vehicle. AMG487 and the same volume of 20% SBE-β-CD solution were injected 2 days before EAP induction.

### The Short Hairpin RNA Mediated Gene Silencing

CXCL10 gene suppression was performed with the short hairpin RNA (shRNA) using adeno-associated virus (AAV) as vector. AAV packaging with the CXCL10 shRNA and shRNA control was purchased from Hanbio, Shanghai, China (contract number HY20200302WY). The target sequence was obtained from previously research and was 5′-TTGATGGTCTTAGATTCCGGA-3′ (26). The AAV-control used the same vector and contained an empty vector backbone. The final titers of AAV-control and AAV-ShRNA-CXCL10 were determined to be 1.6 × 10^12^ vector genomes (vg)/ml and 1.3 × 10^12^ vg/ml, respectively. The detailed protocols referred to previous literature and were described as follows (24, 27). In mice, intravenous injection of AAV containing CXCL10 shRNA or empty vector in a volume of 150 μl/mouse 2 days before EAP induction was performed. On the 14 days after EAP induction, mice were anesthetized with an i.p. injection of pentobarbital sodium (60–80 mg/kg). The lower abdomen was incised to expose the prostate in a sterile condition. The virus solution (AAV-control and AAV-ShRNA-CXCL10) in the micro-injector (Hamilton Co.) was injected slowly into the ventral prostate lobe, dorsolateral prostate lobe, and bilateral coagulating glands with a total volume of 30 μl for each mouse. Then, the wound was closed. Suppression was confirmed using real-time quantitative polymerase chain reaction (RT-qPCR), immunohistochemistry (IHC), and enzyme-linked immunosorbent assay (ELISA) kit for murine CXCL10.

### Behavioral Testing

Pain responses were assessed by tactile allodynia using von Frey force filaments before the mice were sacrificed. As previously described (23, 28), stimulation was confined to the lower abdominal area near the prostate and attention was paid to stimulate different areas within the region to avoid desensitization or “wind up” effects. Briefly, mice were allowed 30 min to acclimate to the new environment in individual plastic chambers with a stainless steel wire grid floor. The tactile allodynia and hyperalgesia were measured using five individual fibers with forces of 0.04, 0.16, 0.4, 1.0, and 4.0 g in every mouse, respectively. Each filament was applied for 1 to 2 s with an interval of 5 s for a total of 10 times. Three types of behaviors were affirmed as positive responses to stimulation: 1) sharp retraction of the abdomen, 2) immediate licking or scratching of the area of filament stimulation, and 3) jumping. The results were presented as percentage of positive responses.

### Histological Evaluation

The prostate tissues were obtained, fixed in 10% neutral formalin, and embedded in paraffin wax. The sections slices (4 μm thick) were stained with hematoxylin and eosin (H&E) and examined by a light microscope for pathological and morphological evaluation. The degree of inflammation was quantified on a four‐point scale from 0 to 3 as previously described (14). The detailed quantitative criteria were shown as follows: 0, no inflammation; 1, mild but definite perivascular cuffing with mononuclear cells; 2, moderate perivascular cuffing with mononuclear cells; and 3, marked perivascular cuffing, hemorrhage, and numerous mononuclear cells in the parenchyma.

### IHC Analysis

The slides of paraffin-embedded tissue specimens were dewaxed, rehydrated, and heated at 100°C for 10 min in citric acid buffer (0.01 M, pH 6.0) for antigen retrieval. The slides were incubated in 3% hydrogen peroxide solution (SP9000; Beijing Zhongshan Jinqiao Biotechnology Co, Ltd.) for 15 min at room temperature and washed in phosphate buffered saline (PBS; pH 7.4) for three times. The slides were blocked with 10% bovine serum albumin. Then, the slides were incubated with anti-CD45 antibody (1:1,000, 20103-1-AP; Proteintech), anti-CXCL10 antibody (1:100, DF6417; Affinity), and anti-CXCR3 antibody (1:100, DF7113; Affinity) overnight at 4°C. After three washes with PBS, the slides were incubated with biotinylated goat anti‐rabbit IgG (1:200) for 2 h at room temperature. Finally, DAB (ZLI-0918, ZSBio, China) was used to detect the immune complexes and the slices were counterstained with hematoxylin. Relative expression levels were analyzed with ImageJ software (National Institutes of Health, Bethesda, MD).

### Immunofluorescence

The slides of paraffin-embedded tissue specimens were dewaxed, rehydrated, and boiled in citric acid buffer (0.01 M, pH 6.0) for antigen retrieval. The slides were permeabilized, blocked, and incubated with the indicated primary antibodies overnight at 4°C. The primary antibodies used were rabbit anti-CXCL10 antibody (NBP2-67004; Novus), rabbit anti-CXCR3 antibody (DF7113; Affinity), rat anti-CD11b antibody (ab8878, Abcam), mouse anti-CD4 antibody (SC-19641, Santa Cruz), mouse anti-*α* smooth muscle actin (*α*-SMA) antibody (SC-53142, Santa Cruz), mouse anti-desmin antibody (SC-23879, Santa Cruz), and mouse anti-Vimentin antibody (SC-6260, Santa Cruz). Subsequently, the slides were incubated with secondary antibodies for 2 h at room temperature. The secondary antibodies used were Cy3 goat anti-rabbit (1:500, A0516, Beyotime), Cy3 goat anti-rat (1:500, A0507, Beyotime), FITC goat anti-mouse (1:500, A0568, Beyotime), and FITC goat anti-rabbit (1:500, A0562, Beyotime). The slides were stained with DAPI and kept in a dark environment. The co-localization of CXCR3 and CXCL10 with other biomarkers was demonstrated by confocal laser scanning microscopy using an Olympus FV3000 microscope.

### ELISA

Cytokine levels in mouse plasma and prostate tissue homogenate samples from immunized mice were detected using ELISA kits for CXCL9 (E-EL-M0020c, Elabscience, Wuhan, China), CXCL10 (E-EL-M0021c, Elabscience, Wuhan, China), CXCL11 (JYM1057Mo, Elisa Lab, Wuhan, China), IFN-γ (E-EL-M0048c, Elabscience, Wuhan, China), IL-6 (CSB-E04639m, Cusabio, Wuhan, China), and MCP1 (CSB-E07430m, Cusabio, Wuhan, China). Serum cytokine levels for CP/CPPS patients were assessed using ELISA kits for CXCL9 (E-EL-H6062, Elabscience, Wuhan, China), CXCL10 (E-EL-H0050c, Elabscience, Wuhan, China), and CXCL11 (E-EL-H0051c, Elabscience, Wuhan, China).

### RNA Isolation and RT-qPCR

Total RNA of prostate tissues was extracted using TRIzol reagent (Thermo, MA, USA) according to the instructions of the manufacturer. The NanoDrop 2000 spectrophotometer (NanoDrop Technologies) was used to measure the purity and concentration of the RNA solution. The reverse transcription reactions were performed using a PrimeScript™ RT reagent kit (Takara, Kusatsu, Japan), and qPCR analysis was prepared at a final volume of 20 µl using a SYBR Green Mix (Takara, Kusatsu, Japan) with primers synthesized by Sangon Biotech (Sangon, Shanghai, China). Primer sequences are listed in **Table S1**. The reactions were measured on an ABI7500 platform (Thermo, MA, USA). The 2^−ΔΔCT^ method was used to determine relative gene expression levels, and GAPDH was used as an internal control to normalize the data. Each reaction was performed in triplicate.

### Western Blotting Assays

For Western blotting assays, the total protein of cells was extracted by radioimmunoprecipitation assay (RIPA) protein lysis buffer (Beyotime Biotech, Jiangsu, China) with a complete protease and phosphatase inhibitor cocktail and PMSF. Protein extract was separated by 12.5% sodium dodecyl sulfate polyacrylamide gel electrophoresis and transferred to polyvinylidene fluoride membranes. The membranes were blocked in 5% non-fat dried skimmed milk for 1 h at room temperature and incubated overnight at 4°C with primary antibodies against Erk1/2 (1:1,000, #4695; CST), p-Erk1/2 (1:1,000, #4370; CST), p38 MAPK (1:1,000, #8690; CST), p-p38 MAPK (1:1,000, #4511; CST), and α-tubulin (1:5,000, AF7010; Affinity). The membranes were washed, incubated with secondary antibodies for 2 h, and visualized using an EZ‐ECL Kit (Biological Industries, Israel).

### Mouse Prostate Dissociation and Flow Cytometry

Mouse prostate dissociation was performed as previously reported (29, 30). Dissected prostates were wash with cold HBSS and cut into 1–2 mm pieces. The pieces of minced prostate were incubated in an enzymatic digestion solution of 4 ml HBSS with 1 mg/ml collagenase I (C0130, Sigma-Aldrich) and 0.2 mg/ml DNase I (Roche, #10104159001) for 40 min at 37°C with continuous shaking. Then, the solutions were filtered through 70 μm filter (Falcon, 352340) and centrifuged at 260*g* for 10 min, and the cell pellet was washed with an additional 10 ml HBSS. Prostatic inflammatory cells were separated by centrifugation in a Percoll density gradient before performing flow cytometry. Fluorescence-conjugated anti-CD11b (BioLegend, 101206), anti-F4/80 (BioLegend, 123116), and Fixable Viability statin 510 (BD Biosciences, 564406) were used to stain cells for 30 min at 4°C. Experiment measurements were performed using CytoFLEX flow cytometer (Beckman Coulter, Brea, CA) and the data were analyzed by FlowJo Software X (Tree Star, Ashland). The detailed operations of loop doors for flow cytometry are shown in **Supplementary Figure S1A**.

### Isolation of Bone Marrow-Derived Macrophages and Cell Treatment

The tibias and femurs of the EAP mice were excised and washed with ice-cold PBS to obtain bone marrow-derived macrophages (BMDMs). The BMDMs were dissociated with ACK lysis buffer for 15 min and washed with PBS once. The BMDMs were cultured in DMEM high glucose medium supplemented with 10% fetal bovine serum and 50 ng/ml macrophage colony-stimulating factor (PeproTech, Rocky Hill, USA) at 37°C and 5% CO_2_ for 1 week. On day 4, the cells were replaced with fresh medium and adherent cells were used for the subsequent experiment. The purity of BMDMs was evaluated using flow cytometry. The purity of the cells is greater than 90% (**Supplementary Figure S1B**).

To detect the effect of CXCL10 on the inflammatory secretion of BMDMs, cells were cultured in serum-free DMEM high glucose medium with or without CXCL10 (100 ng/ml) for 12 h. In inhibiting experiments, BMDMs were pretreated with AMG487 (1 μM) for 1 h and stimulated with CXCL10 (100 ng/ml) for 12 h. In another setting, BMDMs were pretreated with AMG487 (1 μM) for 1 h, with CXCL10 (100 ng/ml) for 12 h, and then stimulated with 50 ng/ml of lipopolysaccharide (LPS, Sigma‐Aldrich, St. Louis, MO) for 3 h. Cells were collected for RT-qPCR analysis and Western blotting assays.

### Migration Assay

Migration assays were performed using 24-well transwell chamber with 8 μm pores (Costar, Bodenheim, Germany) to analyze the effect of CXCL10 on BMDM migration. Amounts of 2 × 105 BMDMs in 200 μl serum-free DMEM high glucose medium were seeded in the upper wells, and 600 μl serum-free DMEM medium with or without CXCL10 (10, 50, 100 ng/ml) was added to the lower wells. In inhibiting experiments, BMDMs were pretreated with AMG487 (1 μM), Erk1/2 inhibitor (PD98059, HY-12028, MedChem Express), or p38 MAPK inhibitor (SB203580, HY-10256, MedChem Express) for 1 h. After 24 h of incubation, cells attached on the lower surface of the membrane were fixed with 100% methanol for 15 min and stained with 0.05% crystal violet staining.

### Chemotaxis Assay

Chemotaxis assays were performed with µ-Slide Chemotaxis (ibidi) following the corresponding protocol. In brief, the cell suspension of BMDMs was washed with PBS and diluted to 3 × 10^6^ cells/ml. The C, D, E, and F ports were closed with plugs and 6 µl of cell suspension was applied onto the filling port (A) of the µ-Slide and 6 µl of air was aspirated from the opposite filling port (B). For the AMG487 group, BMDMs were pretreated with AMG487 (1 μM) for 1 h and were applied onto the filling port (A) of the µ-Slide. The slide was placed in a wet 10-cm Petri dish and transferred to a 37°C incubator until the cells were attached. Then, one of the reservoirs was filled with 65 µl chemoattractant-free medium, and the other was filled with 65 µl chemoattractant (100 ng/ml CXCL10) solution. Once again, all the ports were closed with plugs. Cell migration was recorded by mounting the μ-Slide using Cytation™ 5 instrument with a 37°C incubator and 5% CO_2_. Images were taken for a period of 10 h with frames taken every 10 min. Images were imported as stacks to ImageJ software and analyzed with the manual tracking plug-in. The trajectories and velocities were calculated with the manual tracking feature in chemotaxis and migration tool of ibidi Company.

### Cell Viability Assays

For cell viability assay, 5 × 10^3^ BMDMs were plated in 96-well plates in the presence or absence of CXCL10 (10, 50, 100 ng/ml), AMG487 (1 μM), PD98059 (10 μM), and SB203580 (10 μM) for 1 day. The proliferation rate of cells was detected using a CCK-8 kit (Cell Counting Kit-8, Dojindo Molecular Technology, Japan) according to the instructions of the manufacturer.

### IFN-γ Stimulation

WPMY-1 cell lines were obtained from the Cell Culture Center of the Chinese Academy of Medical Sciences (Shanghai, China). Cells were cultured in DMEM high glucose medium supplemented with 5% fetal bovine serum and were maintained at 37°C with 5% CO_2_. Cells were stimulated with IFN-γ (20 ng/ml), IL-17A (20 ng/ml), and both IFN-γ (20 ng/ml) and IL-17A (20 ng/ml) (PeproTech, Rocky Hill, USA) for 48 h; cells were collected for RT-qPCR; and supernatants were collected for ELISA.

### Statistical Analysis

Statistical analysis was performed by either unpaired, two-tailed Student’s *t*-test, or one-way ANOVA with Bonferroni *post-hoc* test by using the GraphPad Prism version 6.0 software (GraphPad Software, San Diego, CA). Results are expressed as mean ± standard deviation (SD). *P <* 0.05 was considered statistically significant. In the figures, “ns” indicates *P >* 0.05; * indicates *P <* 0.05; ** indicates *P <* 0.01; *** indicates *P < *0.001; **** indicates *P <* 0.0001.

## Results

### Elevated CXCL10 Is Correlated With the Severity of the CP/CPPS Patients

**Figure 1:**
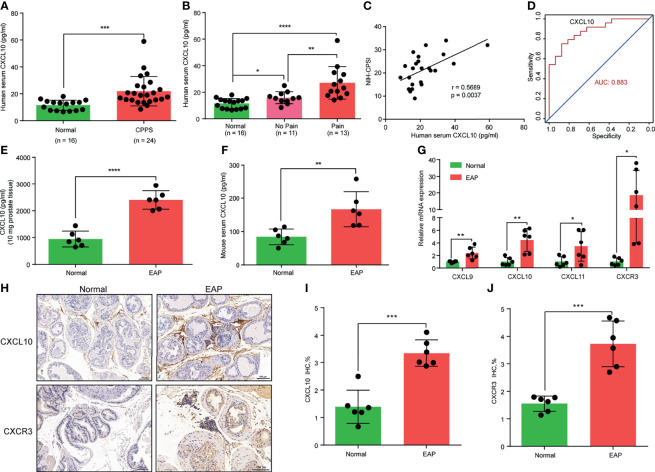
CXCL10 expression is increased in chronic prostatitis patients and experimental autoimmune prostatitis (EAP) mice. **(A)** CXCL10 expression in the serum of chronic prostatitis patients and healthy volunteers. **(B)** CXCL10 expression in the serum of chronic prostatitis patients with and without pain symptom. **(C)** Pearson’s correlation coefficient analysis for CXCL10 expression and National Institutes of Health Chronic Prostatitis Symptom Index. **(D)** The diagnostic efficiency of CXCL10 expression in the serum for chronic prostatitis patients. The expression levels of CXCL10 in prostate **(E)** and serum **(F)** of EAP mice. **(G)** RT-qPCR was used to assess CXCL9, CXCL10, CXCL11, and CXCR3 expressions in prostate from EAP mice. **(H)** IHC staining for CXCL10 and CXCR3 in prostate from EAP mice. Quantification of IHC staining of CXCL10 **(I)** and CXCR3 **(J)** in prostate from EAP mice. Data are shown as mean ± standard deviation (SD) by one-way ANOVA analysis **(B)**, or unpaired, two-tailed Student’s *t*-test analysis **(A, E, F, G, I, J)**, or Pearson’s correlation **(C)**. *P < 0.05; **P < 0.01; ***P < 0.001; ****P < 0.0001.

Cytokine IFN-γ has been postulated to play an important role in the pathogenesis of CP/CPPS patients and the EAP model (17, 18, 31). In the present study, we focused on the roles of IFN-γ-induced chemokines (CXCL9, CXCL10, and CXCL11) in the pathogenesis of CP/CPPS. We compared the expression levels of chemokines CXCL9, CXCL10, and CXCL11 in the serum between CP/CPPS and healthy volunteers. We found significantly elevated levels of CXCL10 in the serum of CP/CPPS patients (**Figure 1A**). Patients with pain symptom have higher expression levels of CXCL10 than those without pain (**Figure 1B**). The expression levels of CXCL10 in serum was positively correlated with the total scores of NIH-CPSI (**Figure 1C**) and could better differentiate CP/CPPS patients from healthy volunteers (**Figure 1D**). The expression levels of CXCL9 showed no significant difference between CP/CPPS and healthy volunteers (**Supplementary Figure S2A**) and between CP/CPPS patients with and without pain (**Supplementary Figure S2B**). The expression levels of CXCL9 in serum was not correlated with the total scores of NIH-CPSI (**Supplementary Figure S2C**) and showed poor diagnostic performance (**Supplementary Figure S2D**). CP/CPPS patients had elevated levels of CXCL11 in serum than healthy volunteers (**Supplementary Figure S2E**). However, patients with and without pain showed no significant difference of CXCL11 levels (**Supplementary Figure S2F**). The expression levels of CXCL11 in serum was not correlated with the total scores of NIH-CPSI (**Supplementary Figure S2G**), but showed general diagnostic performance (**Supplementary Figure S2H**). These data suggest that increased CXCL10 expressions may contribute to the development of CP/CPPS and the expression levels of CXCL10 have potential as a diagnostic marker for CP/CPPS patients.

### CXCL10 Is Overexpressed in the Prostate of the EAP Model

In the EAP model, we first examined the expression levels of chemokines CXCL9, CXCL10, and CXCL11. We found high expressions of CXCL10 in the prostate (**Figure 1E**) and serum (**Figure 1F**) of EAP mice compared with those of the normal group. The expressions of CXCL9 (**Supplementary Figures S2I, J**) and CXCL11 (**Supplementary Figures S2K, L**) were also higher in the prostate and serum of EAP mice by ELISA. RT-qPCR showed significantly increased mRNA levels of CXCL9, CXCL10, and CXCL11 (**Figure 1G**), of which CXCL10 was the mostly increasingly expressed. In addition, we further validated the higher expressions of CXCL10 in the prostate of EAP mice by IHC (**Figures 1H, I**). CXCL10 can bind to CXCR3 to play a role in the accumulation of inflammatory cells (32, 33). Therefore, we also assessed the CXCR3 expression levels in EAP mice. The RT-qPCR (**Figure 1G**) and IHC (**Figures 1H, J**) results showed high-expressed levels of CXCR3 in EAP mice. These results suggest that the CXCL10–CXCR3 axis may be involved in the development of EAP.

### Prostatic Stromal Cell Is a Potential Source of CXCL10

**Figure 2:**
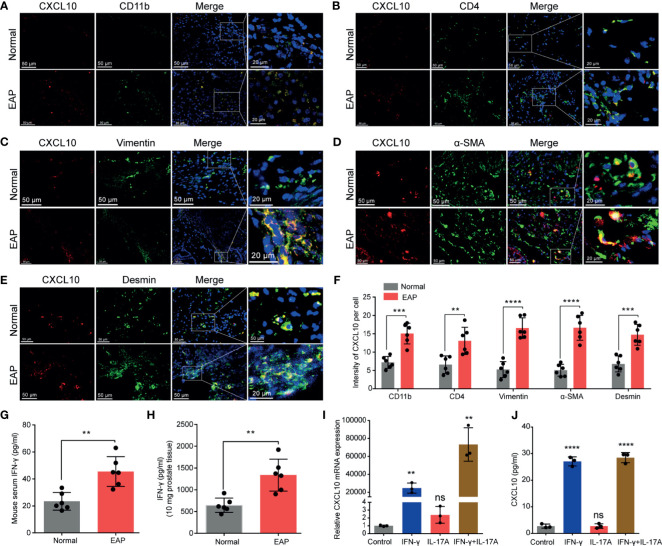
The potential source of CXCL10 in the prostate of EAP mice. Representative photographs of immunofluorescence staining for CXCL10 and markers for CD4+ T cells **(A)**, macrophages **(B)**, and prostatic stromal cells **(C–E)** in the prostate of EAP mice. **(F)** Quantification of CXCL10 immunofluorescence intensity in the experiments of **(A–E)**. High-expressed levels of IFN-γ in the serum **(G)** and prostate **(H)** in EAP mice. The expression levels of CXCL10 in WPMY-1 cells were evaluated with RT-qPCR **(I)** and ELISA **(J)** when treated with IFN-γ, IL-17A, and IFN-γ and IL-17A. Data are shown as mean ± SD by unpaired, two-tailed Student’s *t*-test analysis **(F–H)**, or one-way ANOVA analysis **(I, J)**. “ns” indicates *P > *0.05; **P < 0.01; ***P < 0.001; ****P < 0.0001.

From the results of IHC, CXCL10 was mainly expressed in the prostatic stroma, mainly composed of immune cells and stromal cells (**Figure 1H**). To find out the origin of CXCL10 in the prostate of EAP mice, we performed dual-color immunofluorescence analyses of CXCL10 and a panel of phenotypic cell markers (CD4 for CD4+ T cell; CD11b for macrophage; *α*-SMA, desmin, and vimentin for prostatic stromal cell). CXCL10 was found co-localized with macrophages (**Figure 2A**), CD4+ T cells (**Figure 2B**), and prostatic stromal cells (**Figures 2C–E**). The average fluorescence intensity of CXCL10 in each cell type was also compared in the prostate of EAP mice and that of the normal group (**Figure 2F**). We found a higher intensity of CXCL10 in CD4+ T cell, macrophage, and prostatic stromal cells in the prostate of EAP mice *versus* that of the normal group. Previous studies showed that cytokines IFN-γ and IL-17A are abundant and functional in the prostate and serum of EAP mice (13, 34), which was confirmed in our studies (**Figures 2G, H**). In addition, studies have shown that CXCL10 is secreted by T lymphocytes and monocytes (35). To further confirm whether prostatic stromal cell could secrete CXCL10 in EAP mice, we treated WPMY-1 cells with IFN-γ, IL-17A, and IFN-γ and IL-17A. RT-qPCR and ELISA showed significantly increased expression levels of CXCL10 in IFN-γ, IFN-γ, and IL-17A, but not for IL-17A alone (**Figures 2I, J**). These results suggest that prostatic stromal cell is a potential source of CXCL10 in EAP mice.

### CXCL10 Deficiency Prevents EAP Development by Inhibiting Infiltration of Macrophage to Prostate

**Figure 3:**
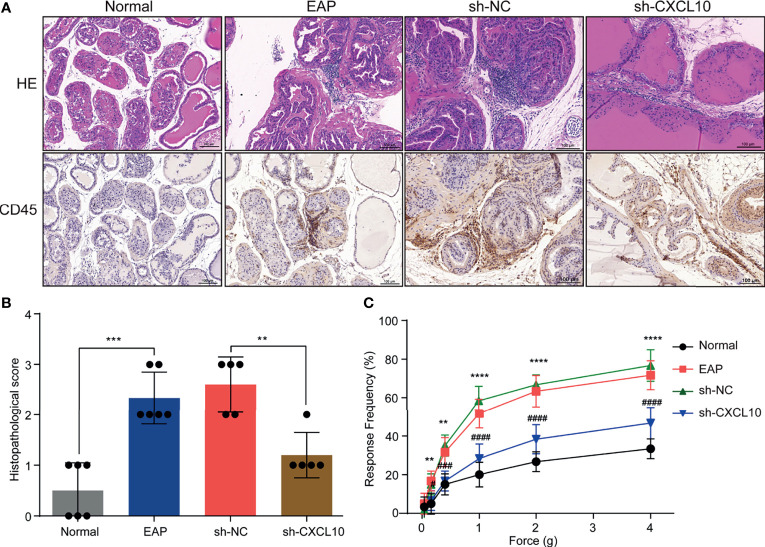
CXCL10 deficiency ameliorates EAP severity. **(A)** Histological evaluation for the degree of inflammation for mice in the normal, EAP, sh-NC, and sh-CXCL10 groups. **(B)** The histopathological scores for mice in the normal, EAP, sh-NC, and sh-CXCL10 groups. **(C)** Pain response test for mice in the normal, EAP, sh-NC, and sh-CXCL10 groups. Data are shown as mean ± SD by unpaired, two-tailed Student’s *t*-test analysis. **P < 0.01; ***P < 0.001; ****P < 0.0001; ^#^ *P <* 0.05; ^###^ *P <* 0.001; ^####^ *P <* 0.0001.

The EAP model was successfully induced with a mixture of PAgs and CFA. The increased inflammatory cells infiltrated in the prostate of EAP mice were confirmed by HE staining and IHC staining for CD45 (**Figure 3A**). The histopathological scores increased in the EAP group compared with those in the normal group (**Figure 3B**). Mechanical stimulation of the pelvic area showed that EAP mice exhibited significantly higher response frequency compared with the normal group, and the response frequency was correlated with the applied force (**Figure 3C**). These results showed that EAP mice were successfully induced. We used shRNA-mediated CXCL10 gene silencing in EAP mice, and the effect of suppression was confirmed using RT-qPCR (**Supplementary Figure S2M**), ELISA (**Supplementary Figure S2N**), and IHC (**Supplementary Figures S2O, P**). HE staining and IHC staining for CD45 showed that the infiltrating inflammatory cells in the prostate of EAP mice were significantly alleviated in the sh-CXCL10 group compared with those in the sh-NC group (**Figure 3A**). The histopathological scores for the sh-CXCL10 group were significantly decreased compared with those for the sh-NC group (**Figure 3B**). The response frequency to mechanical stimulation of the pelvic area in the sh-CXCL10 group was significantly reduced compared with that in the sh-NC group (**Figure 3C**). These results suggested that CXCL10 deficiency ameliorated the inflammatory changes and the pelvic pain of EAP mice.

**Figure 4:**
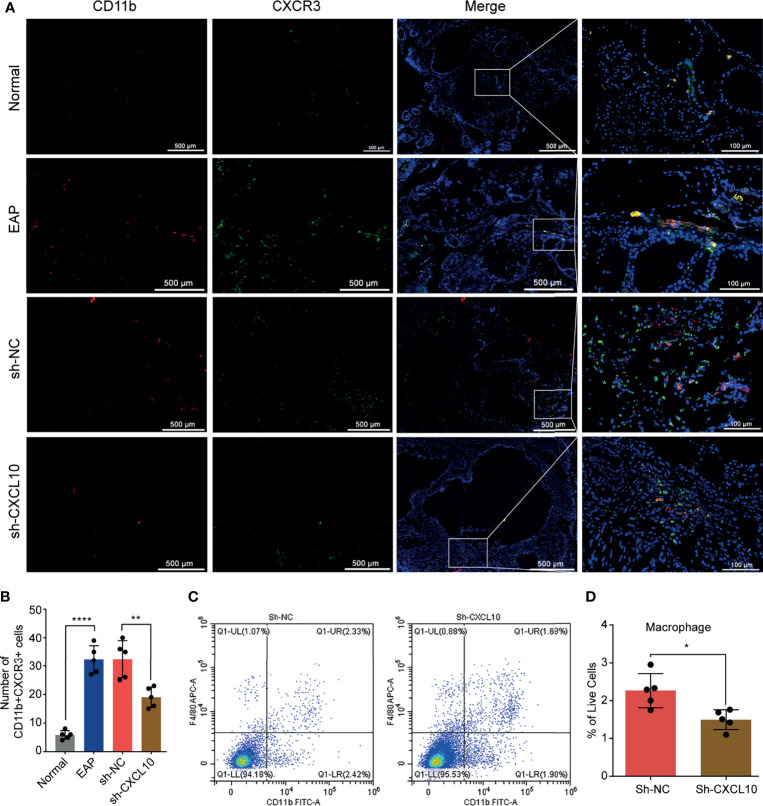
Flow cytometry and immunofluorescence analysis for macrophages in the prostate of EAP mice. **(A)** Representative photographs of immunofluorescence staining of CXCR3 and macrophage markers in the prostate of normal, EAP, sh-NC, and sh-CXCL10 mice. Cells were stained for CD11b (red) and CXCR3 (green). **(B)** The number of CD11b+CXCR3+ cells in the prostate of the normal, EAP, sh-NC, and sh-CXCL10 groups was counted at ×400 magnification. **(C, D)** The results of flow cytometry for macrophages in the prostate of EAP mice. Data are shown as mean ± SD by unpaired, two-tailed Student’s *t*-test analysis. *P < 0.05; **P < 0.01; ****P < 0.0001.

CXCL10 plays roles when binding to CXCR3. Therefore, we performed dual-color immunofluorescence analyses of CXCR3 in EAP mouse. CXCR3 was found co-localized with macrophages (**Figure 4A**). Then, we examined the changes of the numbers of macrophages in prostate. The numbers of dual-positive cells of CD11b and CXCR3 were compared (**Figure 4B**). We found higher numbers of CD11b+CXCR3+ cells in the EAP group compared with those in the normal group (**Figure 4B**). Moreover, the numbers of CD11b+CXCR3+ cells in the sh-CXCL10 group were reduced compared with those in the sh-NC group (**Figure 4B**). The results of flow cytometry showed that the percentage of infiltrating macrophages in the prostate of the sh-CXCL10 group was reduced than that of the control group (**Figures 4C, D**). To exclude the lower percentages of macrophages in the prostate of CXCL10-deficient mice, we simply eliminated the proliferation effect of CXCL10 on macrophages and performed cell viability assays for BMDMs stimulated with different concentrations of rmCXCL10 *in vitro*. We found that CXCL10 does not affect macrophage proliferation (**Figure 6E**). These results suggested CXCL10 deficiency prevents EAP development by inhibiting infiltration of macrophages to prostate.

### Blocking CXCR3 Ameliorates EAP Severity Through Inhibiting Infiltration of Macrophage to Prostate

**Figure 5:**
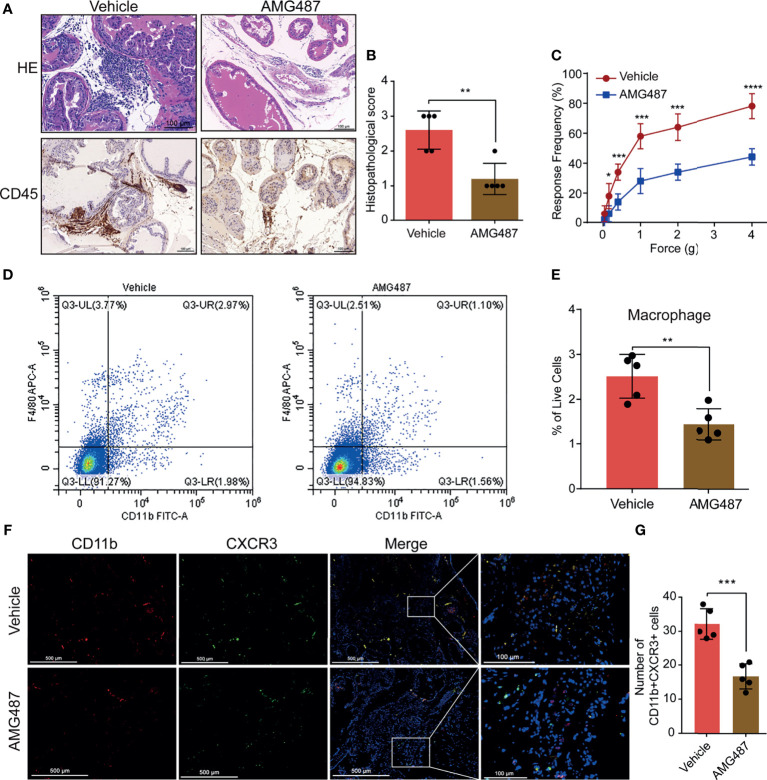
Blocking CXCR3 ameliorates EAP severity through inhibiting infiltration of macrophages to prostate. **(A)** CXCR3 inhibitors (AMG487) ameliorate the inflammation for EAP mice. **(B)** The histopathological scores for the AMG487 and vehicle groups. **(C)** Pain response test for the AMG487 and vehicle groups. **(D, E)** The results of flow cytometry for macrophages in the prostate of the AMG487 and vehicle groups. **(F)** Representative photographs of immunofluorescence staining of CXCR3 and macrophage markers in the prostate of the AMG487 and vehicle groups. Cells were stained for CD11b (red) and CXCR3 (green). The number of CD11b+CXCR3+ cells in the prostate of the AMG487 and vehicle groups was counted **(G)**. Data are shown as mean ± SD by unpaired, two-tailed Student’s *t*-test analysis. *P < 0.05; **P < 0.01; ***P < 0.001; ****P < 0.0001.

To investigate the mechanism underlying CXCL10 regulation of macrophage migration *in vivo*, we used its receptor antagonist (AMG487) to determine whether CXCR3 was involved in CXCL10-induced macrophage migration and inflammation severity of EAP mice. We found significantly reduced infiltrating inflammatory cells in the prostate (**Figure 5A**) and reduced histopathological scores in the AMG487 group than in the vehicle group (**Figure 5B**). The response frequencies to mechanical stimulation of the pelvic area were significantly reduced in the AMG487 group compared with those in the vehicle group (**Figure 5C**). This result indicates that CXCR3 is involved in the development of inflammation and chronic pain in the EAP model of prostatitis.

The macrophages infiltrating in prostate were evaluated using flow cytometry and dual-color immunofluorescence analyses. We found that the numbers of macrophages (**Figures 5D, E**) were reduced in the AMG487 group compared with those in the vehicle group. In addition, the numbers of dual-positive cells of CD11b and CXCR3 in the AMG487 group were reduced compared with those in the vehicle group (**Figures 5F, G**). These results suggested that CXCR3 is involved in the migration of macrophage to prostate in EAP mice.

### CXCL10 Promotes the Migration of Macrophage *via* CXCR3-Mediated Erk and p38 MAPK Activation

**Figure 6:**
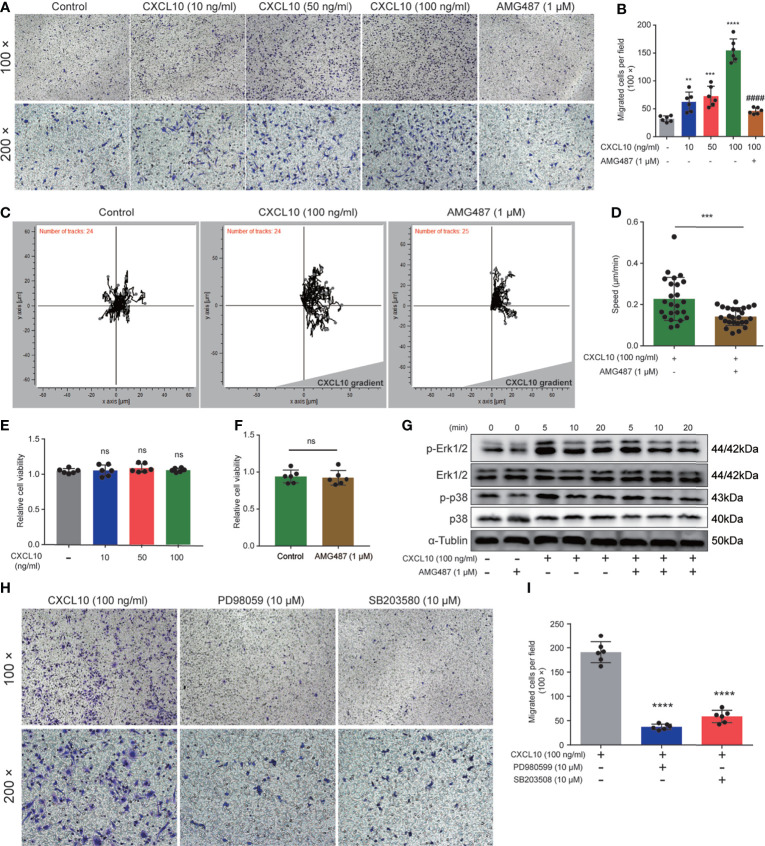
CXCL10 promotes the migration of macrophages *via* CXCR3-mediated Erk and p38 MAPK activation. **(A)** Representative photographs of migration assay for bone marrow-derived macrophages (BMDMs) stimulated with rmCXCL10 (100 ng/ml) for 24 h after pretreating with AMG487 (1 μM) for 1 h. **(B)** The results of migration assay for BMDMs stimulated with rmCXCL10. **(C)** BMDMs were plated on μ-Slide and analyzed for 10 h in the presence of CXCL10 gradient (100 ng/ml). **(D)** Cell speeds were compared for BMDMs between untreated cells and cells pretreated with AMG487 (1 μM) for 1 h. **(E, F)** The viabilities of cells treated with CXCL10 or AMG487 were evaluated with CCK-8 assays. **(G)** Cells were pretreated with AMG487 (1 μM) for 1 h, then with rmCXCL10 (100 ng/ml) for the indicated time, and the phosphorylated p38 MAPK and Erk1/2 levels in BMDMs were detected. **(H, I)** Representative photographs and the results of migration assay for BMDMs stimulated with CXCL10 (100 ng/ml) for 24 h after pretreating with Erk1/2 inhibitor or p38 MAPK inhibitor for 1 h. Data are shown as mean ± SD by one-way ANOVA analysis **(B, E, I)** and by unpaired, two-tailed Student’s *t*-test analysis **(D, F)**. “^####^” indicates *P <*0.0001 compared between the CXCL10 group and the CXCL10 and AMG487 groups. “ns” indicates *P >* 0.05; **P < 0.01; ***P < 0.001; ****P < 0.0001.

Previous studies showed that expressions of CXCR3 on T cells are essential for homing to the prostate gland in EAP mice (18). However, the roles of macrophage expressing CXCR3 in EAP mice remain unclear. Therefore, we next examined the functional consequence of CXCL10–CXCR3 activation for macrophages *in vitro*. First, we test whether CXCL10 could independently trigger macrophage migration. We added rmCXCL10 (10, 50, 100 ng/ml) to the lower chamber, and BMDMs were added to the upper chamber of a transwell device. Results showed that rmCXCL10 could attract BMDMs from EAP mice *in vitro*, and the numbers of migrated BMDMs were correlated with the concentrations of the rmCXCL10 (**Figures 6A, B**). This macrophage-attracting effect of rmCXCL10 was inhibited by AMG487 (1 μM), a CXCR3 receptor antagonist (**Figures 6A, B**). In addition, we used the microfluidics device μ-Slide (ibidi) to detect the role of CXCL10 for the chemotaxis of macrophages. Analysis of the parameters describing single-cell movement found that macrophages exhibited mostly random walk in the absence of CXCL10 (**Figure 6C**). When macrophages were exposed to a stable gradient of CXCL10, they migrated efficiently toward the chemokine source (**Figure 6C**). The chemotactic response of macrophages was also impaired when pretreated with AMG487 for 1 h (**Figures 6C, D**). However, AMG487 (1 μM) showed no effect on cell viability (**Figure 6F**). These results supported the notion that CXCL10 could activate macrophage *via* CXCR3.

Previous studies showed that CXCR3 activation elicited functional effects through Erk1/2 and p38 MAPK phosphorylation (33, 36). Therefore, we determined the protein levels of phosphorylation of Erk1/2 and p38 MAPK in rmCXCL10-activated BMDMs. Our results showed that rmCXCL10 could induce the phosphorylation of Erk1/2 and p38 MAPK within 20 min and with a maximum at 5 min (**Figure 6G**). Moreover, the phosphorylation of Erk1/2 or p38 MAPK was abolished by pretreating BMDMs with CXCR3 inhibitors for 1 h (**Figure 6G**). These results suggested the phosphorylation of Erk1/2 or p38 MAPK was mediated by activation of CXCR3 in BMDMs. To further confirm whether these pathways are required for the CXCL10-mediated migration of macrophage, we pretreated cells with specific inhibitors of Erk1/2 (PD98059) or p38 MAPK (SB203580) for 1 h. We found that incubation with either application of PD980599 or SB203580 could significantly suppress the migration of BMDMs (**Figures 6H, I**). However, both PD980599 (10 μM; **Supplementary Figure S2Q**) and SB203580 (10 μM; **Supplementary Figure S2R**) showed no effect on cell viability. These results suggested that CXCL10 induced the migration of macrophage *via* the interaction of CXCL10–CXCR3 and the activation of the downstream of Erk and p38 MAPK signaling pathways.

### CXCL10 Promotes the Secretion of Inflammatory Mediators of Macrophages *via* CXCR3-Mediated ERK and p38 MAPK Activation

**Figure 7:**
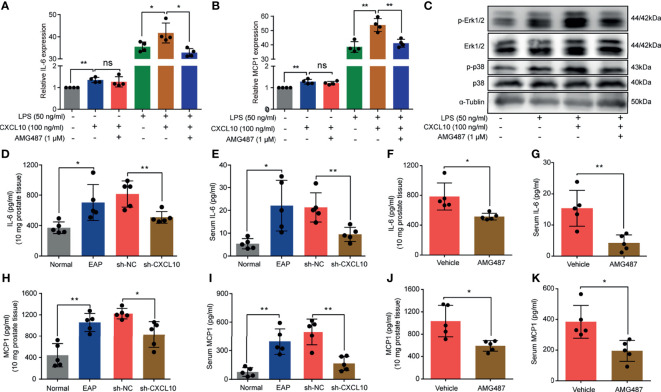
CXCL10 promotes secretions of inflammatory mediators of macrophage *via* CXCR3-mediated ERK and p38 MAPK activation. The mRNA expression levels of IL-6 **(A)** and MCP1 **(B)** in BMDMs were calculated 3 h after LPS treatment. BMDMs from EAP mice were pretreated with AMG487 (1 μM) for 1 h, then stimulated with CXCL10 (100 ng/ml) for 12 h, and then with LPS (50 ng/ml) for 3 h. Cells were collected for detecting the mRNA expression levels of IL-6 **(A)** and MCP1 **(B)**. **(C)** Western blot analysis of the phosphorylation of the ERK1/2, and p38 MAPK signaling pathways in LPS-induced macrophages for 3 h. The expression levels of IL-6 in the prostate **(D)** and serum **(E)** for mice in the normal, EAP, sh-NC, and sh-CXCL10 groups. The expression levels of IL-6 in the prostate **(F)** and serum **(G)** for mice in the AMG487 and vehicle groups. The expression levels of MCP1 in the prostate **(H)** and serum **(I)** for mice in the normal, EAP, sh-NC, and sh-CXCL10 groups. The expression levels of MCP1 in the prostate **(J)** and serum **(K)** for mice in the AMG487 and vehicle groups. Data are shown as mean ± SD by one-way ANOVA analysis **(A, B)** or unpaired, two-tailed Student’s *t*-test analysis **(D–K)**. *P < 0.05; **P < 0.01. ns, no significance.

In addition to induction of the migration of macrophages, whether CXCL10 could independently trigger the production of cytokines was confirmed. BMDMs from EAP mice were stimulated with AMG487 (1 μM) and rmCXCL10 (100 ng/ml) before LPS (50 ng/ml), and the changes of gene expression levels of cytokines were analyzed using RT-qPCR. RT-qPCR measurement for the expressions of inflammatory factors showed that IL-6 and MCP1 were increased after rmCXCL10 treatment with and without LPS for 3 h (**Figures 7A, B**). Pretreatment with AMG487 decreased the expression of IL-6 and MCP1 induced by LPS (**Figures 7A, B**). These results suggested CXCL10 could promote secretions of IL-6 and MCP1 of macrophages *via* CXCR3. To further confirm whether CXCR3-mediated Erk and p38 MAPK activation is involved in the production of inflammatory mediators, we determined the protein levels of phosphorylation of Erk1/2 and p38 MAPK in LPS-activated BMDMs. We found that LPS improved the phosphorylation of Erk1/2 and p38 MAPK, and rmCXCL10 pretreatment significantly enhanced the phosphorylation of Erk1/2 and p38 MAPK. On the other hand, AMG487 pretreatment significantly inhibited the phosphorylation of Erk1/2 and p38 MAPK (**Figure 7C**). These results suggested that CXCL10 promoted the secretion of inflammatory mediators of BMDMs *via* CXCR3-mediated ERK and p38 MAPK activation *in vitro*.

To further address this aspect, we examined the expression changes of IL-6 and MCP1 *in vivo*. The expression levels of IL-6 in prostate and serum were increased in EAP mice, and CXCL10 deficiency could reduce the expressions of IL-6 in prostate (**Figure 7D**) and serum (**Figure 7E**). The expression levels of MCP1 were increased in EAP mice, and CXCL10 deficiency could reduce the expressions of MCP1 in prostate (**Figure 7H**) and serum (**Figure 7I**). The roles of CXCR3-mediated pathways in the secretion of inflammatory mediators were confirmed. We found that the expression levels of IL-6 in prostate (**Figure 7F**) and serum (**Figure 7G**) were reduced in the AMG487 group. In addition, the expression levels of MCP1 in prostate (**Figure 7J**) and serum (**Figure 7K**) were also reduced in the AMG487 group. These results suggested the important roles of CXCR3-mediated pathways in the secretion of inflammatory mediators.

## Discussion

In the present study, we showed that CXCL10 was upregulated in the serum of CP/CPPS patients and was associated with the severity of the CP/CPPS patients. CXCL10 could promote the migration of macrophages and secretion of inflammatory mediators *in vivo* and *in vitro* to contribute to the pathogenesis of EAP. CXCL10 deficiency ameliorates EAP severity by inhibiting CXCR3-mediated Erk and p38 MAPK activation. In addition, we further demonstrated that CXCL10 is co-localized with infiltrated CD4+ T cells, macrophage, and intrinsic prostatic stromal cells. Prostatic stromal cells were considered as one of the contributors to CXCL10 in the pathogenesis of EAP.

CP/CPPS, a poorly understood clinical syndrome, was characterized by local signs and symptoms of chronic inflammation and discomfort in the pelvic region lasting longer than 3 months in the absence of identifiable urogenital infections (37). The methods for the diagnosis of CP/CPPS mainly depend on the symptoms of patients, and biomarkers used for clinical diagnosis are very limited. Recently, several biomarkers in expressed prostatic secretion (EPS), urine, semen, or serum have been proposed for the diagnosis of CP/CPPS (7, 38–40). Levels of urinary prostatic exosomal protein have been suggested as a novel biomarker for the diagnosis for CP/CPPS and can be used as a sign of the severity of CP/CPPS (41). Penna et al. quantified several cytokine and chemokine levels in seminal plasma of CP/CPPS patients and revealed that IL-8 in seminal plasma was significantly elevated in CP/CPPS patients and was positively correlated with symptom score. IL-8 is a reliable biomarker for CP/CPPS patients and can discriminate CP/CPPS IIIA *versus* IIIB (41). Watanabe et al. showed that nerve growth factor (NGF) level in the prostatic fluid of CP/CPPS patients is correlated with symptom severity and response to treatment (42). NGF could be used as a new biomarker to evaluate the symptoms of CP/CPPS and the effects of treatment (42). Wei et al. showed that soluble B7-H3 level in EPS is a novel chronic prostatitis marker that correlates negatively with symptom score (43). In addition, several inflammatory cytokines in serum or seminal plasma were elevated, including macrophage migration inhibitory factor, IL-1β, TNFα, IL-6, IL-17, IFN-γ, and MCP1 (5, 31, 41, 44). Nonetheless and to the best of our knowledge, we report for the first time elevated levels of CXCL10 in the serum from CP/CPPS patients. CXCL10 has been reported to be involved in cancer-induced bone pain (45). In the present study, CXCL10 concentration in serum was correlated directly with the pain of CP/CPPS patients. These results showed that CXCL10 might contribute to the pathophysiology of CP/CPPS. In addition, CXCL10 concentration in serum can discriminate CP/CPPS patients from healthy volunteers, suggesting that CXCL10 could be used as a biomarker for the diagnosis and evaluation of the symptoms of CP/CPPS.

Previous studies have supported a key role of IFN-γ in the pathogenesis of the disease in the EAP model (5, 17, 18). Chemokines CXCL9, CXCL10, and CXCL11, which were induced by IFN-γ, were almost null in IFN-γ knockout NOD mice with absent leukocyte infiltration in the prostate (18). These results suggested that chemokines CXCL9, CXCL10, and CXCL11 might be involved in the pathogenesis of EAP. Most studies link CXCL10 expression to the recruitment of T cells. Chemokine CXCL10 is a powerful recruiter of Th1 cells expressing CXCR3 into target tissues, which is responsible for organ-specific autoimmune diseases (19). Chemokine CXCL10 has been predicted to play an important role in leucocyte recruitment and immune response in a number of inflammatory and autoimmune diseases (16, 32, 33, 46). Studies suggested that IFN-γ could induce the expression of CXCR3 on T cells, which was the common receptor of chemokines CXCL9, CXCL10, and CXCL11 and was essential for migrating to the prostate gland (18). However, the roles of these chemokines in the pathogenesis of EAP and specific mechanisms remain unclear. The role of CXCL10 in macrophage recruitment has been reported as well (33, 46). Macrophages are key factors in the development of CP/CPPS (15, 47). However, little is known about the effect of CXCL10 on macrophage function in the EAP model. In this study, we show that CXCL10 could induce the migration of macrophage into prostate in the EAP model *in vivo*. The migration assay of macrophages *in vitro* further confirmed the chemotaxis of CXCL10. Moreover, the CXCL10-induced chemotaxis for macrophage is not only concentration dependent but also associated with the activation of the Erk1/2 and p38 MAPK pathways. These migrated macrophages can promote the progression of EAP by releasing proinflammatory mediators.

It has been shown that inflammatory cytokines, including IFN-γ, IL-6, and MCP1, were increased in CP/CPPS patients and in the EAP model (5, 11, 41). The reduction of serum IL-6 was correlated with the release of clinical symptoms, suggesting that IL-6 played important roles in the development of CP/CPPS (11). In the present study, stimulation of cultured BMDMs from the EAP model with rmCXCL10 induces IL-6 expression, whereas the rise of IL-6 levels in the prostate of EAP models is less intense in CXCL10-deficient mice. These results indicated that CXCL10 could regulate the expressions of proinflammatory cytokine IL-6 in macrophages involved in the pathogenesis of EAP. It is known that MCP1 could modulate macrophage migration and has been identified as a prominent modulator of inflammatory immune microenvironment in several inflammatory diseases (48). Recent studies showed that MCP1 is an essential mediator for the inflammation of prostate tissue and pelvic pain in the EAP model (49). Studies have shown the interactions between CXCL10 and MCP1 (50). In the present study, we showed that CXCL10 could induce the expressions of MCP1 of macrophage *in vivo* and *in vitro*. The increased MCP1 could attract more macrophages to infiltrate the prostate, forming a positive feedback loop to aggravate prostatitis. In addition, we demonstrated that CXCL10 induced the expressions of inflammatory mediators in macrophage *via* phosphorylation of Erk and p38 MAPK, which is mediated by the CXCL10 receptor, CXCR3.

At present, the involvement of prostatic stromal cells or epithelial cells in CP/CPPS is still controversial. Histopathological features of CP/CPPS patients and the EAP model suggested infiltration of inflammatory cells in prostatic stroma (5, 12). These inflammatory cells could produce cytokines and growth factors to regulate cell growth through either paracrine or autocrine pathways. It has been shown that cytokines including IL-8, IL-2, IL-4, and IFN-γ and culture supernatant of mast cells are able to induce growth of prostatic stromal cells (51). However, whether prostatic stromal cells could induce and sustain chronic inflammatory processes in CP/CPPS pathogenesis remains unclear. Previous studies showed that CP/CPPS was an inflammatory process, with prevalent prostatic stromal involvement (52). Increased cytokines in benign prostatic hyperplasia (BPH) could induce BPH cells to produce inflammatory mediator, including IL-6, IL-8, and CXCL10 to create a positive feedback loop that can amplify inflammation (53). Prostate stromal cells incubated with the culture supernatant of mast cells could express CXCL8 and CCL2 to amplify inflammation (54). However, the roles of prostatic stromal cells in the pathological process of CP/CPPS remain unclear. In the present study, we showed that CXCL10 was co-located with prostatic stromal cells in EAP mice. In addition, we found the expression levels of CXCL10 were significantly increased when stimulated with cytokines IFN-γ and IL-17A, all of which are abundant and functional in the prostate of EAP mice. These results suggest that prostatic stromal cells might be involved in the pathogenesis of EAP. Prostatic stromal cells were able to induce and sustain chronic inflammatory processes by producing CXCL10 and creating a positive feedback loop that can amplify inflammation.

**Figure 8:**
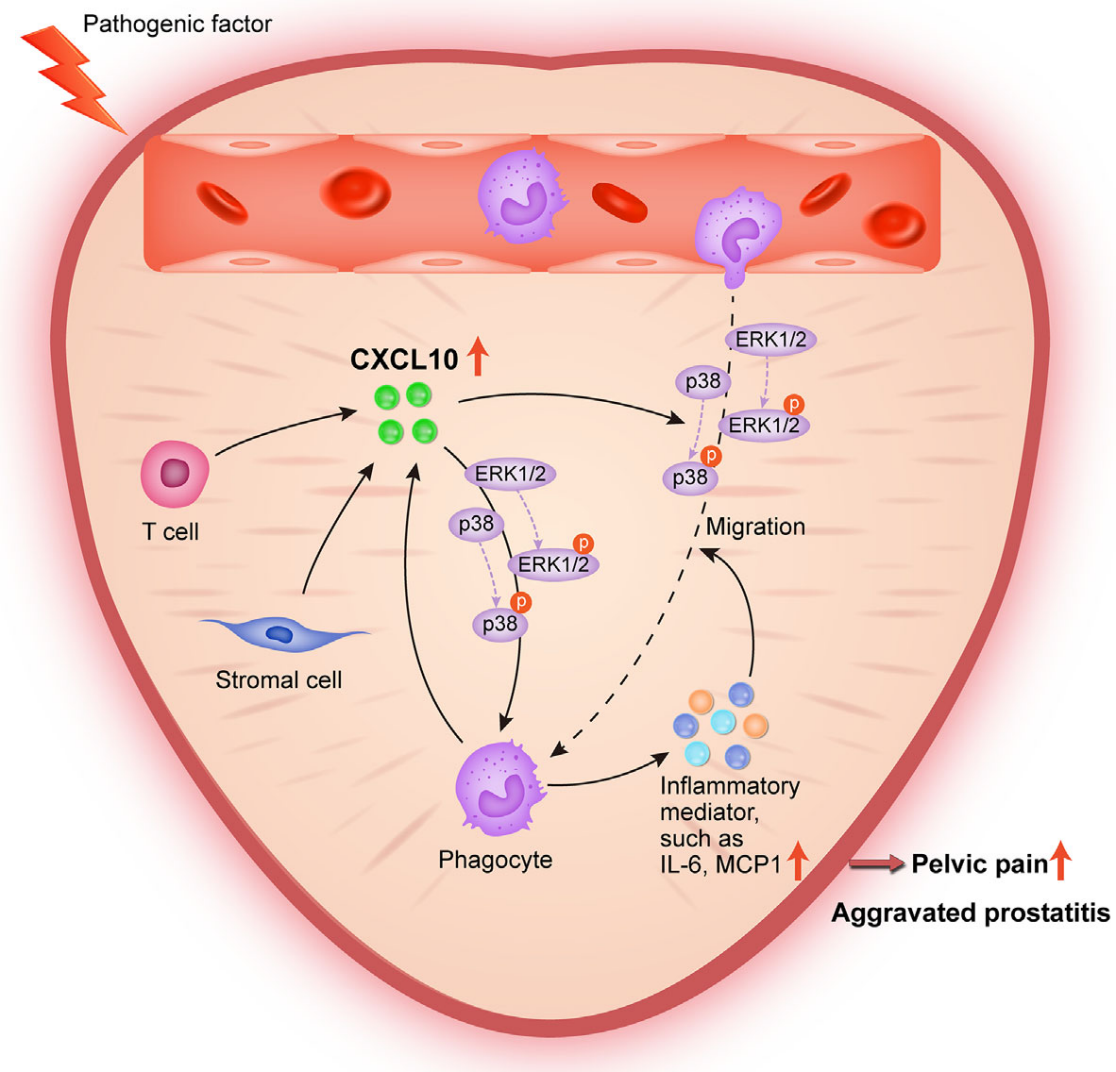
Schematic diagram of the pathogenic roles of CXCL10 in EAP. During EAP, CXCL10 recruits macrophages to prostate and induces the secretions of proinflammatory mediators *via* the Erk1/2 and p38 MAPK signaling pathways, leading to the exacerbation of EAP. Prostatic stromal cells, T cells, and macrophages are potential sources of CXCL10.

In conclusion, the present study provides lines of evidence that CXCL10 promotes the migration of macrophages and the secretion of inflammatory mediators *via* CXCR3-mediated ERK and p38 MAPK activation, leading to the pathogenesis of EAP. During pathologic progression of prostatitis, prostatic stromal cells and infiltrating leukocytes can also serve as the source of CXCL10, forming a positive feedback loop to amplify inflammation (**Figure 8**). Our results identify CXCL10 as an important mediator of prostatitis involved in inflammatory infiltration and pain symptoms, which could serve as a potential therapeutic target and diagnostic marker for CP/CPPS. However, an additional clinical trial is needed to confirm clinical application values.

## Data Availability Statement

The original contributions presented in the study are included in the article/**Supplementary Material**. Further inquiries can be directed to the corresponding authors.

## Ethics Statement

The studies involving human participants were reviewed and approved by the ethical committee of the First Affiliated Hospital of Anhui Medical University. The patients/participants provided their written informed consent to participate in this study. The animal study was reviewed and approved by the Committee for Animal Care and Use of the Animal Center of Anhui Medical University.

## Author Contributions

CL, LZ, and XC conceived and designed the experiments. XH, SG, MZ, and FM performed the experiments and analyzed the data. LGZ and JZ helped with the animal experiments. MZ, FM, JZ, ST, and CY helped in obtaining the clinical samples. XH wrote the manuscript. ST and CY checked the manuscripts. All authors contributed to the article and approved the submitted version.

## Funding

This study was funded by the National Natural Science Foundation of China (Nos. 81630019, 81870519), Anhui Natural Science Foundation (2108085QH315), Scientific Research Foundation of the Institute for Translational Medicine of Anhui Province (No. 2017ZHYX02), and Research Fund of Anhui Institute of Translational Medicine (No. ZHYX2020A003).

## Conflict of Interest

The authors declare that the research was conducted in the absence of any commercial or financial relationships that could be construed as a potential conflict of interest.

## Publisher’s Note

All claims expressed in this article are solely those of the authors and do not necessarily represent those of their affiliated organizations, or those of the publisher, the editors and the reviewers. Any product that may be evaluated in this article, or claim that may be made by its manufacturer, is not guaranteed or endorsed by the publisher.

## Supplementary Material

Supplementary Figure S1 Flow cytometry analysis. **(A)** The detailed operations of loop doors for flow cytometry. **(B)** The purity of bone marrow-derived macrophages.

Supplementary Figure S2 The expression changes of CXCL9 and CXCL11 in chronic prostatitis patients and experimental autoimmune prostatitis (EAP) mice. CXCL9 **(A)** and CXCL11 **(E)** expressions in serum for chronic prostatitis patients and healthy volunteers. CXCL9 **(B)** and CXCL11 **(F)** in serum for chronic prostatitis patients with and without pain symptom. Pearson’s correlation coefficient analysis for CXCL9 **(C)** and CXCL11 **(G)** expressions and National Institutes of Health Chronic Prostatitis Symptom Index. The diagnostic efficiency for CXCL9 **(D)** and CXCL11 **(H)** expressions in serum for chronic prostatitis patients. The expression levels of CXCL9 in prostate **(I)** and serum **(J)** of EAP mice. The expression levels of CXCL11 in prostate **(K)** and serum **(L)** of EAP mice. The effects for shRNA-mediated CXCL10 gene silencing in EAP mice were confirmed using RT-qPCR **(M)**, ELISA **(N)**, and IHC **(O)**. Quantification of IHC staining of CXCL10 for mice in sh-NC and sh-CXCL10 group **(P)**. The effects on cell viability were evaluated with CCK8 assays for PD98059 **(Q)**, and SB203580 **(R)**. Data are shown as mean ± SD by one-way ANOVA analysis **(B, F)**, or unpaired, two-tailed Student’s *t*-test analysis **(A, E, I–N, P–R)**, or Pearson’s correlation **(C, G)**. “ns” indicates *P >* 0.05; **P <* 0.05; ***P <* 0.01; ****P <* 0.001; *****P <* 0.0001.
