# Supplementary figures and images for: Pathogenic Roles of CXCL10 in Experimental Autoimmune Prostatitis by Modulating Macrophage Chemotaxis and Cytokine Secretion
Source: Front Immunol. 2021 Sep 29;12:706027. doi: 10.3389/fimmu.2021.706027 (PMC8511489; doi:10.3389/fimmu.2021.706027)

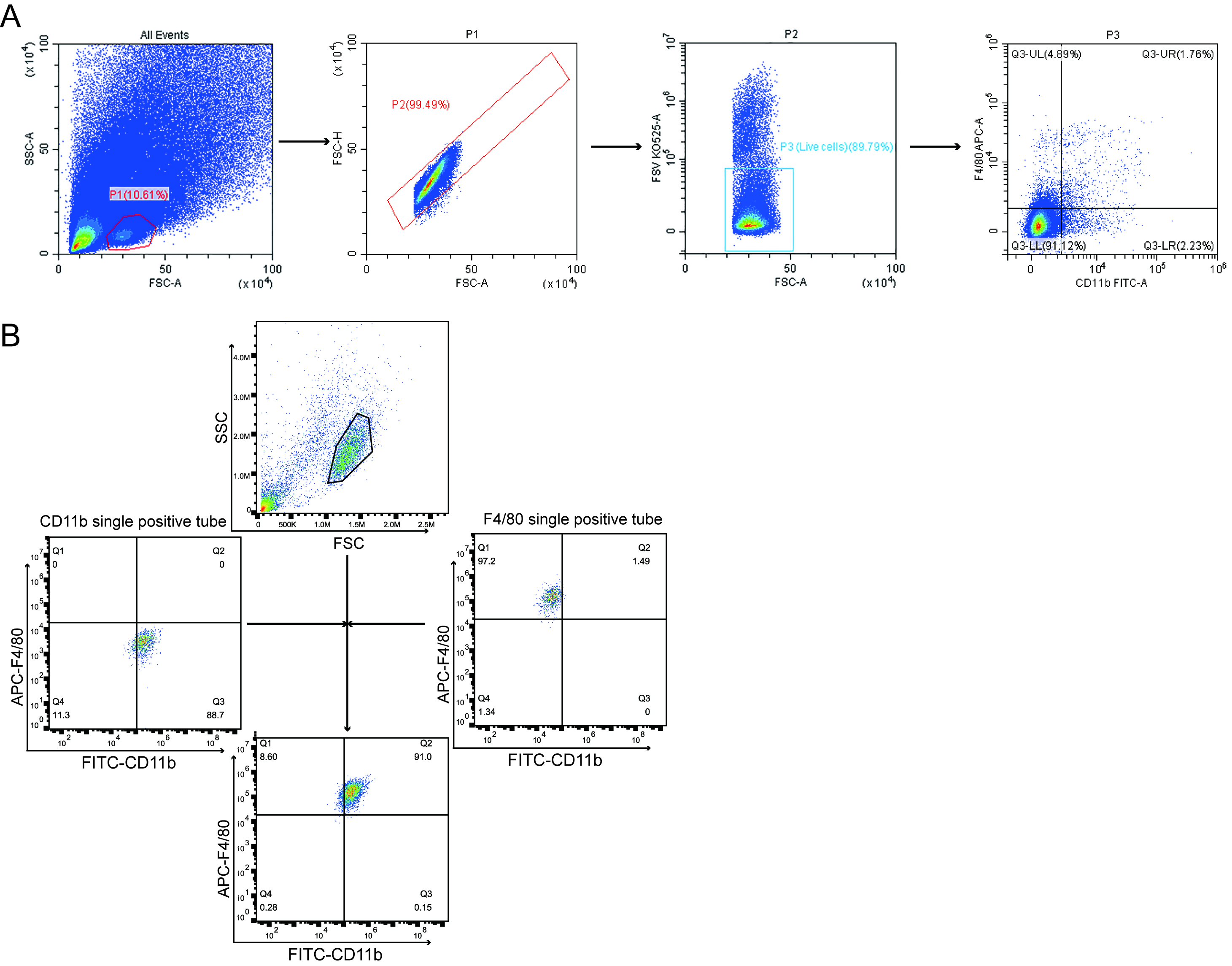

Supplement: Supplementary Figure S1 — Flow cytometry analysis. (A) The detailed operations of loop doors for flow cytometry. (B) The purity of bone marrow-derived macrophages. [file Image_1.tif]

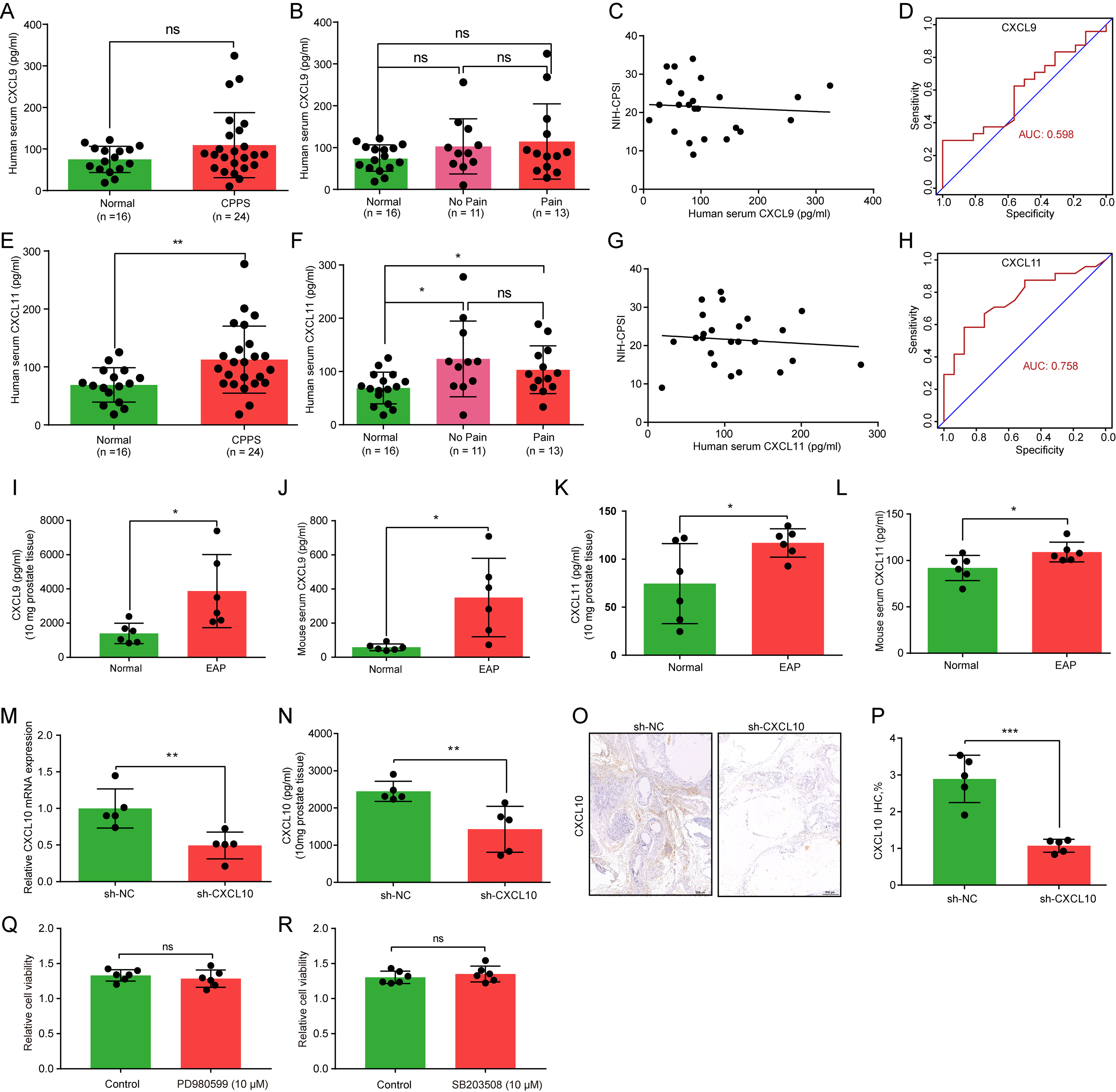

Supplement: Supplementary Figure S2 — The expression changes of CXCL9 and CXCL11 in chronic prostatitis patients and experimental autoimmune prostatitis (EAP) mice. CXCL9 (A) and CXCL11 (E) expressions in serum for chronic prostatitis patients and healthy volunteers. CXCL9 (B) and CXCL11 (F) in serum for chronic prostatitis patients with and without pain symptom. Pearson’s correlation coefficient analysis for CXCL9 (C) and CXCL11 (G) expressions and National Institutes of Health Chronic Prostatitis Symptom Index. The diagnostic efficiency for CXCL9 (D) and CXCL11 (H) expressions in serum for chronic prostatitis patients. The expression levels of CXCL9 in prostate (I) and serum (J) of EAP mice. The expression levels of CXCL11 in prostate (K) and serum (L) of EAP mice. The effects for shRNA-mediated CXCL10 gene silencing in EAP mice were confirmed using RT-qPCR (M), ELISA (N), and IHC (O). Quantification of IHC staining of CXCL10 for mice in sh-NC and sh-CXCL10 group (P). The effects on cell viability were evaluated with CCK8 assays for PD98059 (Q), and SB203580 (R). Data are shown as mean ± SD by one-way ANOVA analysis (B, F), or unpaired, two-tailed Student’s t-test analysis (A, E, I–N, P–R), or Pearson’s correlation (C, G). “ns” indicates P > 0.05; *P < 0.05; **P < 0.01; ***P < 0.001; ****P < 0.0001. [file Image_2.tif]
