# Supplementary material for: Pathogenic Roles of CXCL10 in Experimental Autoimmune Prostatitis by Modulating Macrophage Chemotaxis and Cytokine Secretion
Source: Front Immunol. 2021 Sep 29;12:706027. doi: 10.3389/fimmu.2021.706027 (PMC8511489; doi:10.3389/fimmu.2021.706027)
Supplement: Supplementary file 3 [file Table_1.docx]

Table S1. Primers for real-time quantitative polymerase chain reaction.

| **Symbol** | **Forward primer** | **Reverse primer** |
| --- | --- | --- |
| Mouse |  |  |
| CXCL9 | 5’-GAGCAGTGTGGAGTTCGAGG-3’ | 5’-TCCGGATCTAGGCAGGTTTG-3’ |
| CXCL10 | 5’-GGATGGCTGTCCTAGCTCTGTA-3’ | 5’-CTTAGAACTGACGAGCCTGAGC-3’ |
| CXCL11 | 5’-TGTAATTTACCCGAGTAACGGC-3’ | 5’-CACCTTTGTCGTTTATGAGCCTT-3’ |
| CXCR3 | 5’-TACCTTGAGGTTAGTGAACGTCA-3’ | 5’-CGCTCTCGTTTTCCCCATAATC-3’ |
| IL-6 | 5’-CTGCAAGAGACTTCCATCCAG-3’ | 5’-AGTGGTATAGACAGGTCTGTTGG-3’ |
| MCP1 | 5’-GCTACAAGAGGATCACCAGCAG-3’ | 5’-GTCTGGACCCATTCCTTCTTGG-3’ |
| GAPDH | 5’-TGACCTCAACTACATGGTCTACA-3’ | 5’-CTTCCCATTCTCGGCCTTG-3’ |
| Human |  |  |
| CXCL10 | 5’-AAGGATGGACCACACAGAGG-3’ | 5’-AGCAGGGTCAGAACATCCAC-3’ |
| GAPDH | 5’-GGGAGCCAAAAGGGTCAT-3’ | 5’-GAGTCCTTCCACGATACCAA-3’ |
